# Retention of doctors in remote, rural and First Nations communities using distributed general practice education: a scalable solution

**DOI:** 10.3389/fmed.2025.1584501

**Published:** 2025-05-09

**Authors:** Patrick Giddings, Belinda O’Sullivan, Matthew McGrail

**Affiliations:** ^1^Remote Vocational Training Scheme Ltd., Albury, NSW, Australia; ^2^Rural Clinical School, University of New South Wales, Albury, NSW, Australia; ^3^Rural Clinical School, University of Queensland, Rockhampton, QLD, Australia; ^4^Monash Rural Health, Monash University, Bendigo, VIC, Australia

**Keywords:** remote supervision, professional support, general practice, distance education, workforce retention, health equity, First Nations, rural health

## Abstract

The value of distributed training of the medical workforce is well documented. Australia’s Remote Vocational Training Scheme (RVTS) provides a scalable approach to specialist training in general practice that utilizes distance education and remote supervision. RVTS enables trainees to stay in their rural, remote and First Nations communities while working toward specialist certification as a general practitioner. The program, which supports both international and domestically trained graduates through tailored supervision and education, has operated across Australia for 25 years. Trainees are supported both professionally and socially over 4 years. An independent evaluation (2023–24) demonstrated a 78% completion rate among participants who remained in the same rural or remote practice for an average of 5.2 years. Two years after completing the program, 49% were still working in the community where their training commenced, well above documented retention benchmarks for these settings. High levels of participant satisfaction were reported, ranging from 88 to 100% across various indicators. The evaluation found that the program supports retention by eliciting five participant responses: comfort, confidence, competence, belonging, and bonding. Engagement and connection between participants are maintained through accessible technology, real-time support, virtual small-group learning, and twice-yearly in-person workshops. Despite the program’s focus on high-need areas, it is cost-effective compared to similar rural training schemes. The experience of RVTS can inform other countries seeking to enhance rural workforce retention, particularly for underserved populations and migrant healthcare workers. The adaptable structure of the program aligns with the global development goals of the World Health Organization.

## 1 Introduction

Evidence supports the value of distributed training of the medical workforce. It can improve service delivery and patient satisfaction at the training sites, increase opportunities for clinical teaching at rural locations and provide economic benefits to rural regions (1–5). However, most of the evidence is based on medical schools using face-to-face modes of supervision and teaching involving rural immersion across a broad region. There are fewer examples of distributed general practice training that use remotely delivered distance education and supervision to train continuously in the same rural or remote location. In this perspective, we seek to describe Australia’s Remote Vocational Training Scheme (RVTS program), which has been operating in Australia for 25 years (6). It uses distance education and remote supervision to assist non-specialist doctors already working in remote, rural and First Nations communities to stay in the same community while they train to become General Practitioners (GPs) (6). In doing so it targets workforce retention and service continuity while extending opportunities for trainees to complete their GP qualifications.

Australia’s national health insurance scheme, Medicare, requires general practitioners to hold formal specialist certification in general practice. Exemptions to this requirement exist in designated areas of workforce shortage, predominantly located in rural and remote regions. Under these provisions, many medical practitioners including both domestic and doctors trained overseas who are not yet certified as specialist GPs in Australia, often take up general practice roles in rural areas, where they can access different programs that provide appropriate supervision. One of these programs, which also provides GP training in eligible locations, is the RVTS (see Box 1).

**Box 1** Australia’s health context.

Australia is vast, spanning more than 7.7 million square kilometers. It encompasses a wide range of geographical diversity, including temperate areas, tropical rainforests, coastal environments, arid areas, and deserts (7).

The population exceeds 26 million, with approximately 72% residing in major cities, primarily located along the eastern coast. The remaining 28% live outside the major cities, spread across more than 12,300 rural localities covering 99.3% of Australia’s land area (8). First Nations people represent 3.8% of the overall population but account for a significantly higher proportion in many remote and rural locations (9). Inland areas are sparsely populated. Access to healthcare outside of major centers can be challenging, complicated by distance and health workforce shortages.

Australia is a parliamentary democracy that is a federation of 6 states and two territories with three tiers of government: Federal, State/territory and Local. The federal government is responsible for national issues such as foreign policy and defense as well as the universal health scheme Medicare. States and Territories deliver services such as education, police, and public hospitals, while local governments look after municipal and community-based services. The federal system allows for regional variations to respond to local needs (10).

The healthcare system includes both public and privately funded services. Medicare is a universal public insurance scheme that provides free or subsidized access to general practice and specialist services outside of public hospitals. Public hospitals provide free inpatient care and are funded by the states (11). Rural and Remote populations with lower access to services utilize proportionately less Medicare funds (12).

Australian Medical School training takes 5–6 years for undergraduates and 4 years for post-graduate courses (13). This is followed by a one-year internship, then a residency, and finally, specialty training. General practice is delivered in accordance with the training standards of either the Royal Australian College of General Practitioners or the Australian College of Rural and Remote Medicine, spanning 3 or 4 years, leading to registration as a specialist general practitioner. The Australian Government funds trainees in the Australian General Practice Training Program (AGPT) and RVTS (14, 15).

In 2025, 1,504 trainees commenced in the AGPT and 32 in RVTS, while a further 100 commenced in a specific rural generalist pathway (16). Workforce maldistribution impacts rural and remote health outcomes. The Australian Government addresses the problem by supporting rural training pathways such as RVTS, offering financial incentives for rural practice, and expanding telehealth services.

We draw on evidence from an independent evaluation of the RVTS program, conducted in 2023–24, to describe the program’s design and its achievements (6). We then explore how the program could serve as a model for other countries considering similar initiatives, while also addressing some of the potential challenges associated with broader adoption.

## 2 RVTS design and delivery

RVTS Ltd. is an independent training provider responsible for the national delivery of the RVTS program (6). The RVTS selects non-specialist doctors who are supported in their progression toward specialist qualifications in general practice and rural generalist medicine. The curriculum and specialist certifications are accredited by the Australian Medical Council through two independent medical colleges concerned with the specialty of general practice, the Royal Australian College of General Practitioners (RACGP) and the Australian College of Rural and Remote Medicine (ACRRM). The RVTS is an accredited provider for the Colleges and addresses all the same training standards of specialist GP training, through a flexible delivery model. What sets RVTS apart from other GP training programs in Australia is its focus on keeping trainees working in the rural and remote communities where they are based. As such, it is primarily a workforce retention program which uses education support to help non-specialist doctors achieve GP specialist qualifications in Australia. Unlike most other specialist training programs, RVTS requires participants to remain in the same location for most of the training (6). RVTS-eligible practices are in towns with populations of 15,000 or fewer, or within Aboriginal Medical Services that support rural First Nations communities. Many of these sites are small, underserved communities facing persistent workforce shortages and high staff turnover, often relying on locum tenens to maintain service delivery (17). General practitioner turnover is particularly high in these communities, with an average retention of approximately 2 years, incurring an estimated cost of A$74,000 per departing GP (18). Halving turnover and reducing reliance on locum services in remote areas could yield annual savings of up to A$32 million in one state alone (19) and enhance access to high-quality, relationship-based care (20) with lower all-cause mortality (21).

The communities targeted by the RVTS often place a high value on hosting GP trainees but face significant barriers to doing so. These include limited supervisory capacity and challenges in attracting suitable trainees (22, 23). Although these communities may have limited clinical resources, they often offer valuable opportunities for extended scope of practice, enabling rich learning opportunities to support progression toward specialist qualifications in general practice (22, 24). The RVTS program also works with other agencies to help communities with the highest needs to access non-specialist doctors who will stay, supported with high quality remote supervision and distance education (33). This helps the community to gain from a trainee who is embedded in a co-learning community of practice, progressively developing skills as part of specialist training, while remaining employed in the same community (25). This in turn delivers a more stable, skilled and locally engaged workforce which is critical for high quality care for rural and First Nations communities (25).

The program is led from a regional location (Albury, New South Wales) and was conceptualized by a team of experienced rural GPs and rural generalists informed by the insights of a pilot project in its early stages. It has grown gradually since it commenced in 2000, targeting the enrolment of 32 trainees annually (6). Currently this quota consists of 22 trainees in general practice or rural generalist medicine based in general practices in small and medium rural towns of 15,000 population or less, and 10 working in rural Aboriginal Medical Services, serving First Nations communities.

The program works hard to engage suitable candidates in the distributed locations. This involves regularly liaising with communities, the state and territory governments, rural workforce agencies, medical colleges and other programs, to facilitate engagement. The selection process seeks to offer places to trainees who are likely to be safe and able to reflect and learn effectively while continuing their practice and training under a remote supervision and distance education mode of delivery.

Most participants are International Medical Graduates (IMGs) (6). Australia mandates this group to work in rural areas for up to 10 years upon arrival to help to fill rural workforce shortages and Australia has increasing reliance on this group with greater rurality (26, 27). IMGs bring diverse credentials and prior clinical experiences, which can add significant value to primary healthcare in rural and First Nations communities (6). RVTS’ requirements of staying in the same practice while completing specialist training aligns with preferences of many IMGs who often seek stability after experiencing frequent relocation, changes in employers and inconsistent supervision (28).

With ongoing participation contingent on remaining in the same location, the RVTS is purposefully designed as an end-to-end vocational training program based in a single location. It includes tools, resources and processes to support and develop skilled GPs who are retained in that location. While the RVTS program delivers education and training remotely, its strategic focus on retention also means that it gives strong attention to the wider professional and social supports trainees need to remain resilient and continue working in this context. This includes matters such as career coaching, real-time support and help with risk mitigation around issues in the practice. The nature and depth of this support is significant given the pressures trainees face when working and living in remote, rural and First Nations communities, including the idea that urban practice is superior, so-called geographical narcissism (29).

The RVTS mostly supports community-based training outside of hospital settings. Supervisors and medical educators are qualified GPs and rural generalists, experienced in small rural practices, based in locations distant to the trainees but generally in the same region. They commonly support up to two trainees continuously over the course of the program. Each participant is paired with a consistent supervisor and medical educator. Regular in-person visits are undertaken for direct observation, supplementing the online learning and feedback (30). Having been in operation for 25 years, the RVTS program has recruited many past trainees (who make up around 30% of its current supervision cohort) as the next generation of medical educators and supervisors. This group comes with strong experience, commitment and empathy for the training body. They also align with the goals of the program making it comfortable for trainees to reach out for advice (30).

The remote supervision and distance education is delivered using simple, adaptable, off-the-shelf technology systems, of both voice and video, through one-on-one and small group learning (30). Regular supervision meetings occur at pre-set frequencies and can be increased via phone, email and face-to-face visits according to participant needs so there is confidence that help is there when it is needed. RVTS participants also receive additional explicit layered teaching and supervision to build the skills required for remote, rural and First Nations communities, without assuming any prior knowledge (30). This includes support for communication, culturally responsive care, healthcare systems and relevant skills. This approach helps to standardize foundational skills, to create a safe environment for asking a wide range of questions and to ensure participants feel confident and competent to practice safely in rural and remote Australia. Trainees also access a suite of online educational resources that can be tailored or scaled to meet individual trainee needs (30). Additionally, peer engagement is fostered through a WhatsApp group and twice-yearly multi-day face-to-face workshops, with families funded to attend. These gatherings help to cultivate a strong sense of belonging and bonding among trainees and their families. As trainees work in more remote areas, they become part of a community of practice that respects and values the work of rural and remote doctors and their importance to the health of rural communities. This experience supports their sense of being valued and recognized, culminating in Australian specialist qualifications in general practice or rural generalist medicine. More information about the RVTS training model is available online (30).

## 3 The benefits of remote supervision and distance education

Building on four formative evaluations completed in 2001, 2003, 2005 and 2015, a 2023–24 independent evaluation led by the University of Queensland explored the RVTS program outcomes of candidate satisfaction, achievement of specialist certification and longer-term retention for all cohorts. The evaluation was undertaken with ethical approval (University of Queensland Human Research Ethics Committee; 2023/HE001926, 24 October 2023), and the results were reported in a series of five publications in 2024 (6, 25, 31–33). The results compared favorably with benchmarks which were established by a Project Reference Group and informed by the expectations of the program management team, and from expectations of satisfaction and retention shown for this context reflected in the broader literature. These benchmarks were had been set before commencing the evaluation (to reduce bias). The benchmarks, classified as high, medium and poor performance, specifically tailored for a GP training cohort, including IMGs, based in remote, rural and First Nations settings, reflecting the unique context in which the RVTS program operates.

At the time of the evaluation, RVTS had enrolled 506 participants. Since 2013, 82% of the enrolled group were IMGs, mostly from countries where English is not the primary language (6). The IMG cohort had a median total of 14 years clinical experience before commencing, while IMGs and domestic trainees had 5 and 6 years, respectively, working as registered medical practitioners before commencing training (6). The participants covered 350 communities, 83% of which were small rural (under 5,000 population) or remote towns and 15% were medium-sized rural towns of 5–15,000 population (6). Additionally, 65% of the communities were located inland, more than 50 km from the coast. At the time of the evaluation, 101 participants were still actively enrolled, 317 had completed the program, and 88 had withdrawn, giving a program completion and specialist certification rate of 78%. More information about participants is available elsewhere (6).

RVTS participants had worked for a mean of 1.6 years in the same practice before they joined the program (31). They participated in the program for a mean of 3.6 years (excluding those still active), resulting in a total mean of 5.2 years in the same practice location (31). This is approximately 3 times longer than the expected term of retention period for doctors in areas of this rurality (18). Within 2 years of completing the program, 49% of the participants remained in the same practice location, exceeding the high performing benchmark of 30% (31). The mean long-term annual retention rate was 33% in rural and remote regions, surpassing the high performing benchmark of 30 and 50% in all rural areas exceeding the high performing benchmark of 40% (31). These results exceeded the expected turnover rates of general practitioners in more remote areas, noting that IMG cohorts are also more challenging to retain. More information about retention outcomes is available (31). Annual satisfaction survey results demonstrated high-performing satisfaction levels ranging from 88 to 100% across various measures, exceeding the high performing benchmark of 75% (34).

The drivers of the strong satisfaction and retention outcomes were explored as part of a nested realist evaluation within the broader evaluation project (32). This articulated a theory about how the bundled interventions within the RVTS program stimulate five responses in participants: comfort, confidence, competence, belonging, and bonding. In turn, these responses addressed the professional and non-professional needs of participants to promote satisfaction and retention (32). The remote supervision and distance education mode of delivery helped these interventions reach the target audience in a cost-effective way. This suggests that remote learning and distance education systems should avoid being driven by technology but rather should be driven by purpose, which, in the case of the RVTS, has been GP workforce retention.

Unpublished data from the evaluation also identified the RVTS program as cost-effective. The true cost per RVTS participant per year (targeting rural and remote regions and First Nations communities) was comparable to the cost of other rural-focused GP training while noting that RVTS trains a higher proportion of IMG candidates who train in more distributed locations for longer than was observed for other rural training programs for general practice (16). The return on investment is enhanced by the gains in quality of GP-led care from the pre-, during, and post-program retention years for the three-to-four-year cost of supporting participants in the RVTS program.

## 4 Applying the learning to other countries

The RVTS program is relatively small in the whole scheme of GP training in Australia, however, there are no other GP pathways focused specifically on the retention of trainees in remote, rural and First Nations settings. As such, the RVTS holds a unique place in Australia’s rural health workforce environment, a position reinforced by the positive evaluation findings. However, more broadly, the program has the potential to inform remote supervision and distance education for general practice workforce retention in similar contexts in other countries. Some countries are already pursuing distributed GP training and could adapt some of the RVTS approach to enhance retention goals (35). Other countries may be considering developing something like the RVTS program (36). In either scenario, other countries and regions are welcome to explore partnerships with the RVTS for mentorship, advice and resource sharing. This partnership could be facilitated through groups like rural WONCA and primary healthcare training bodies in all World Health Organization regions, as an extension of their work on building rural pathways (37).

For many low- and middle-income countries, managing the costs and resources for sustaining rural training pathways are a major barrier to implementation (37). The cost of the RVTS program is primarily driven by the expenses associated with specialist general practice training in Australia, including supervision, assessments, training materials, and compliance with program accreditation requirements. Therefore, costs in other countries may vary depending on the type of qualifications pursued and the expenses related to delivering educational materials and processes via a remote model. Additional expenses include the cost of using accessible technology for communication between participants and staff, maintaining a secure database for storing trainee information, and providing educational materials. Additionally, there are staffing costs. Operationally, the RVTS program has established a central and virtual hub of around 35 part–time staff equivalent to 18 full-time positions. This includes program administration and management staff, supervisors and medical educators. Depending on the resources available to interested countries or communities, the face-to-face workshops that fund the travel and accommodation for participants and their families could be replaced with other strategies that are more affordable. Critically, it is important to consider whether alternative strategies could achieve the same impact of belonging and bonding as is achieved by the RVTS workshops, for a cohort of isolated trainees working in challenging conditions. The workshops are important for doctors and their families to feel socially valued, to build connections and take a much-needed break from their usual environment, to recharge socially (32).

Many of the tools, resources and processes used by the RVTS program could be adaptable and applicable to the education and technology architecture of other countries. For example, much of the communication across the network can happen by telephone email and free online chat sites. Nevertheless, the digital divide that some rural areas experience is an important consideration for planning remote supervision and distance education programs (38). Within the context of Australia’s vast geography and dispersed remote and rural communities, basic communication technologies are considered suitable because they are common and familiar to RVTS participants.

The current RVTS program targets participants who are already working relatively independently and bring prior skills and clinical experience to their locations. However, given the program’s high satisfaction rates, specialist certification and long-term retention outcomes, there is potential to learn from the program and explore how it might be refined or adapted for other medical trainees or health workers such as rural or remote nurses (39). One of the strengths of the RVTS program is that it includes cultural considerations and diversity within its design (adaptable to a wide range of doctors of different backgrounds) (39). If the RVTS program were to be adapted for less experienced trainees, it might require more intensive face-to-face support at the outset, along with more frequent face-to-face events throughout the year, such as four workshops instead of the current two. Expanding distributed education and remote supervision models may become increasingly important when supervision capacity declines in more distributed locations (40).

RVTS is a mature program, and its published outcomes help reinforce confidence in its effectiveness. Since the RVTS targets non-specialist doctors already based in the communities of interest, with a primary focus on retention, its outcomes are not directly comparable to other distributed rural training, which involve rotations or short-term immersions into different communities (6). More specific and tailored program designs aimed at recruiting and retaining health workers already based in rural areas could be applied within rural policy and initiatives. This is directly linked to the potential community benefits of enhanced continuity of care, the presence of health workers who can support team building and contribute to broader quality improvement within health services (25). Promoting retention is particularly warranted in smaller rural, remote and First Nations communities, who are the most underserved and face high costs associated with workforce (17). By focusing on non-specialist doctors already based in rural areas, the RVTS program has naturally drawn a significant number of IMGs who are required to work in rural and remote settings. The RVTS model can inform the WHO Global Code of Practice on the International Recruitment of Health Personnel (41). This Code calls on countries to ethically support migrant workers by providing them with access to education and professional development to the same standards offered to the domestic workforce. However, many countries lack a practical model for extending education and professional support to this group, particularly if they are located in rural areas.

In summary, the RVTS is a mature, evidence-based program that employs distance education and remote supervision. It has been fully described and evaluated, demonstrating strong, long-term results. They highlight the importance of designing programs focused on retention in challenging contexts, emphasizing the need to address factors that support not only training but also the overall resilience of health workers. The RVTS program offers valuable insights for informing the development of rural health workforce policy and initiatives aimed at achieving distribution and retention of a skilled workforce to meet the needs of remote, rural and First Nations communities.

## Data Availability

The data analyzed in this study is subject to the following licenses/restrictions: data is available on request to the authors subject to ethical approval. Requests to access these datasets should be directed to giddings@rvts.org.au.
